# Theoretical Exploring Selective-Binding Mechanisms of JAK3 by 3D-QSAR, Molecular Dynamics Simulation and Free Energy Calculation

**DOI:** 10.3389/fmolb.2020.00083

**Published:** 2020-05-27

**Authors:** Jingyu Zhu, Qianqian Yu, Yanfei Cai, Yun Chen, Hui Liu, Wenqing Liang, Jian Jin

**Affiliations:** ^1^School of Pharmaceutical Sciences, Jiangnan University, Wuxi, China; ^2^Hubei Key Laboratory of Agricultural Bioinformatics, College of Informatics, Huazhong Agricultural University, Wuhan, China; ^3^CF PharmTech Inc., Suzhou, China

**Keywords:** JAK3 inhibitor, selectivity, 3D-QSAR, CoMFA, CoMSIA, molecular dynamics simulation, free energy calculation

## Abstract

Janus kinase 3 (JAK3) plays a critical role in the JAK/STAT signaling pathway and has become an attractive selective target for the treatment of immune-mediated disorders. Therefore, great efforts have been made for the development of JAK3 inhibitors, but developing selective JAK3 inhibitors remains a great challenge because of the high sequence homology with other kinases. In order to reveal the selective-binding mechanisms of JAK3 and to find the key structural features that refer to specific JAK3 inhibition, a systematic computational method, including 3D-QSAR, molecular dynamics simulation, and free energy calculations, was carried out on a series of JAK3 isoform-selective inhibitors. Necessary pharmacodynamic structures and key residues involved in efficient JAK3-inhibition were then highlighted. Finally, 10 novel JAK3 inhibitors were designed, the satisfactory predicted binding affinity to JAK3 of these analogous demonstrated that this study may facilitate the rational design of novel and selective JAK3 inhibitors.

## Introduction

Cytokines play important roles in multiple cellular functions, such as cell proliferation, invasion, survival, inflammation, and immunity. Cytokines are therefore critical regulators of immunological diseases and cancers (Lin and Karin, 2007). Accumulative studies show that many cytokines including interleukins (ILs), growth factors, and interferons (IFNs) play a critical role in the triggering of inflammatory reactions through the JAK-STAT (Janus kinase – signal transducers and activators of transcription) pathway, whose excessive expression is always detected in areas that are inflamed (Danese et al., 2015). The JAK family is a class of non-receptor tyrosine kinases and can be divided into four members, JAK1, JAK2, JAK3, and TYK2 (tyrosine kinase 2). They act as hubs during the signaling transduction process for multiple cytokines (Imada and Leonard, 2000; Leonard and Lin, 2000; Shuai and Liu, 2003), and therefore depicts JAKs as attractive targets of immunosuppression. The JAK-STAT pathway is initiated when cytokines bind with their respective type I or II receptors, followed by the phosphorylation of specific JAKs, receptors, and STATs, thereby transducing signals to the nucleus (O’Shea et al., 2004; Ghoreschi et al., 2009; O’Shea and Plenge, 2012). Among the JAK family, JAK1/2 and TYK2 are expressed ubiquitously in many tissues and organs, whereas JAK3 is limited to hematopoietic, myeloid, and lymphoid cells (Kawamura et al., 1994). This distinguishing feature makes JAK3 expression and function restricted to the immune system. The restricted expression pattern can avoid the risk of undesirable side effects when inhibiting the JAK3 pathway. Therefore, JAK3 is considered to be a relevant and ideal drug target for immunosuppression and opens the door for the development of more JAK3-specific inhibitors. The story of JAK3 selective inhibitors started after the development of Tofacitinib – a pan-JAK inhibitor that was the first FDA-approved JAK inhibitor for the treatment of RA (rheumatoid arthritis). Tofacitinib was originally developed as a JAK3 selective inhibitor, but it finally turned out to be a pan-JAK inhibitor (Flanagan et al., 2010; Knapp et al., 2012; Thoma et al., 2014). After that, a large number of JAK3 inhibitors have been developed and reported over the last couple of decades, but up to now, only three JAK3-specific inhibitors have entered clinics: Peficitinib, Decernotinib, and PF-06651600 (Anniina et al., 2019). Among them, PF-06651600 displays the most salient selectivity due to the covalent, irreversible interaction with the Cys909 of JAK3, which is a unique residue within the JAK family (Farmer et al., 2015; Thorarensen et al., 2017; Hamaguchi et al., 2018), and at present, it is a mainstream approach in the design of JAK3 selective inhibitors. Another approach is to develop ATP-competitive inhibitors with reversible binding interactions with specific regions in JAK3. Compared with non-covalent drugs, covalent drugs have stronger electrophilicity and often bind irreversibly to the target (Juswinder et al., 2011). As a result, once the miss-target effect occurs, many adverse and even toxic reactions emerge leading to tissue damage or an immune response *in vivo*. Therefore, it would be more preferable to discover a non-covalent JAK3 inhibitor.

Since residues around the binding pocket are highly conservative in these JAK members, it is naturally a hard challenge to discover an inhibitor specifically targeting JAK3 isoform (Clark et al., 2014). A proposed and generally accepted strategy to achieve this goal would be to employ *in silico* methods, such as quantitative structure-activity relationship (QSAR) analysis, molecular docking, molecular dynamics (MD) simulations, and free energy calculations, etc. Therefore, in this paper, a series of potent JAK3 inhibitors reported by Soth et al. (2013) were collected to investigate the mechanisms of JAK3 binding selectivity through an integrated computational strategy. 3D-QSAR models with CoMFA (Comparative Molecular Field Analysis) and CoMSIA (Comparative Molecular Similarity Indices Analysis) were first built to probe the structural features of the inhibitors with a view to general structure-activity relationships. Then MD simulation and free energy calculations were employed to identify the pivotal interaction and hot residues, which are the key to JAK3 selective binding. Finally, 10 new JAK3 inhibitors were designed according to the simulation results and the inhibitor with the best-predicted potency was taken as a reference to investigate the JAK3-inhibiting selectivity.

## Materials and Methods

### Dataset

A dataset of a total of 73 JAK3 inhibitors with satisfactory pharmacokinetic profiles was obtained from four studies in the literature (Jaime-Figueroa et al., 2013; Lynch et al., 2013; Soth et al., 2013; de Vicente et al., 2014). The bio-affinities of these inhibitors cover a range of 4 orders of a magnitude and are evenly distributed over this range. These molecules were constructed based on the structure of compound 61 (Cpd61) retrieved from the co-crystallized structure of the Cpd61/JAK3 complex (PDB ID: 3ZC6), and then optimized with MMFF94 force filed in SYBYL-X2.0. Before the performance of QSAR analysis, the reported half maximal inhibitory concentrations (IC_50_) of these inhibitors were all transformed into pIC_50_ (-logIC_50_) as dependent variables. The structures and biological activities of these compounds are listed in Supplementary Table S1. The dataset was then randomly divided into the training set and the testing set through the *Generate Training and Test Data* module in Discovery studio 3.5 (DS3.5), and the ratio of the training set (56 inhibitors) to the test set (17 inhibitors) is ∼3:1 (the test set molecules labeled with asterisk in Supplementary Table S1).

### 3D-QSAR Model Building

As we know, the high quality of QSAR models relies heavily on reasonable structural alignment (Li et al., 2019). Thus, Cpd61 with the highest bioactivity was stretched from the crystal structure (PDB ID: 3ZC6) and chosen as the reference molecule. All inhibitors were then aligned over a common pyrrolopyrazine core (shown in Supplementary Figure S1). The CoMFA model was built by placing the aligned molecules in the 3D cubic lattice with a regularly spaced grid of 2.0 Å. The standard Tripos steric and electrostatic fields using sp^3^ carbon probe atom with a + 1 charge and a van der Waals radius of 2.0 Å, and the default settings with the 30 kcal/mol cutoff were used. In addition, an “*Advanced CoMFA*” module containing H-bond fields and indicator fields were both investigated, and the cutoff values applied were also 30 kcal/mol. Additionally, CoMSIA was carried on and the molecular alignment was placed in a 3D grid similar to that of CoMFA. Five molecule force fields were calculated with a C + probe atom in a default grid spacing of 2.0 Å, namely, steric, electrostatic, hydrophobic, hydrogen bond acceptor, and donor fields. The remaining parameters were set as default.

To assess the reliability and predictive ability of the developed QSAR models, internal and external validations were carried out, respectively. The optimum number of components (NOC) is determined through PLS (Partial least squares) analysis using the leave-one-out (LOO) cross-validation method. The result of the cross-validation is described as *q*^2^ and it is recognized to possess the ability of internal prediction when its value is between 0.5 and 1.0. Based on the known NOC, non-cross-validation was implemented and correlation coefficient *r*^2^, standard error (SEE), and *F*-test value were yielded. Usually, A model with *q*^2^ > 0.5, *r*^2^ > 0.9, *F* > 100, and SEE < 0.3 is considered acceptable. In order to evaluate the predictive ability of the generated models, a representative test set was used to estimate the *r*^2^*_*pred*_* (*r*^2^*_*predicted*_*). The predicted coefficient should be close to 1, which represents a good external predictive ability of models.

### Molecular Dynamics Simulation

Two complexes (PDB ID: 3ZC6, 4HVI) derived from the RSCB Protein Data Bank were used as initial structures for MD simulation with the *SANDER* program of the AMBER18 software package (Case et al., 2005). The general AMBER force field (GAFF) (Junmei et al., 2004) was employed on the ligands and the Amber ff14SB (Hornak et al., 2010) was used for the proteins. Each inhibitor was optimized with the semi-empirical AM1 method in Gaussian09 (Stewart, 2004). The complexes were placed in an octahedron water box with a cutoff value of 10 Å in all directions and in a TIP3P solvation environment. The particle mesh Ewald (PME) method (Essmann et al., 1995) was applied to estimate long-distance electrostatics. And the system charge was neutralized by adding Na^+^ ions (Hess and Nf, 2006). For energy minimization, we first performed steepest descent followed by conjugate gradients for the relaxation of the system (Zhu et al., 2019a,b,c,d). Secondly, the whole system was heated, and the temperature rose from 0 to 300 K gradually under NVT and following NPT, equilibration at 1atm was conducted. Finally, a 200-ns MD simulation was implemented on the equilibrated system under 300 K, 1 bar pressure. The temperature and pressure of the system were controlled by the Langevin dynamics method (Somedatta and Sanjoy, 2013). The SHAKE algorithm (Ryckaert et al., 1977) was used to constrain all the hydrogen atoms. After the MD simulation, the trajectories were extracted and analyzed, the root-mean-square deviation (RMSD) was calculated to assess the stability of the complex systems.

### Binding Free Energy Analysis

The snapshots of each system obtained from the last 100-ns stable MD trace file were used for further binding free energy calculations with the MM/GBSA (Molecular Mechanics/Generalized Born Surface Area) method (Lyne et al., 2006) in AMBER18. And the equations for the calculation of ΔG_bind_ were displayed as follows:

(1) $$\Delta \,G_{\text{bind}}={\text{G}}_{\text{complex}}-\left({\text{G}}_{\text{receptor}}+{\text{G}}_{\text{ligand}}\right)=\Delta \,H-T\,\Delta \,S$$

(2) $$\Delta \,H=\Delta \,E_{\text{MM}}+\Delta \,G_{\text{sol}}-T\,\Delta \,S$$

(3) $$\Delta \,E_{\text{MM}}=\Delta \,E_{\text{ele}}+\Delta \,E_{\text{vdw}}+\Delta \,E_{\text{internal}}$$

(4) $$\Delta \,G_{\text{sol}}=\Delta \,G_{\text{GB}}+\Delta \,G_{\text{GS}}=\Delta \,G_{\text{GB}}+\gamma \,\text{S}\,\text{A}\,\text{S}\,\text{A}+\beta$$

The binding free energy is the sum of the enthalpic contribution (ΔH) and the entropy contribution (−TΔS). The enthalpy ΔH involves the intermolecular energy (ΔE_*MM*_) and the solvation free energy (ΔG_*sol*_). The former represents the gas-phase interaction energy in the complex, and it consists of the electrostatic (ΔE_*ele*_), van der Waals (ΔE_*vdw*_), and internal energy (ΔE_*internal*_). The latter is composed of the polar electrostatic solvation energy contribution (ΔG_*GB*_) and the non-polar contribution (ΔG_*GS*_). Classical statistical thermodynamics were used to calculate the TΔS with the normal-mode program. A set of snapshots were extracted from the last equilibrated MD trajectory for the calculation of the ΔG_*bind*_.

Energy contribution decomposition on each residue was also estimated with the MM/GBSA approach in order to identify which residue plays a critical role in the binding with inhibitors. The interaction of each residue with the inhibitor is decomposed to four elements as shown in the following equation:

(5) $$\Delta \,G_{\mathrm{inhibitor}-\mathrm{residue}}=\Delta \,G_{\text{vdw}}+\Delta \,G_{\text{ele}}+\Delta \,G_{\text{GB}}+\Delta \,G_{\text{SA}}$$

Where the van der Walls (ΔG_*vdw*_) and electrostatic (ΔG_*ele*_) interactions constitute the interaction between the inhibitor and protein in the gas phase. ΔG_*GB*_ and ΔG_*SA*_ represent the polar and non-polar contributions constituting the solvation free energy. The generalized Born (GB) model is employed to calculate ΔG_*GB*_ (Onufriev et al., 2000), and the ΔG_*SA*_ is calculated on the basis of SASA using the ICOSA technique (Gohlke et al., 2003). The decomposition calculations are all performed based on the same snapshots studied previously.

### Molecular Docking

In order to reveal the binding patterns of the newly-designed inhibitor, D9 was docked into JAK1/2/3, respectively. The crystal structure of JAK1 (PDB ID: 6N7A), JAK2 (PDB ID: 4IVA), JAK3 (PDB ID: 4HVI) retrieved from RCSB Protein Data Bank were used as receptors for subsequent docking simulations. First, three isoforms were prepared with the *Prepare Protein* module and D9 was sketched with DS3.5 and was then prepared with the *Prepare Ligand* module. Second, the *CDOCKER* module in DS3.5 was employed to perform docking with default parameters set. Finally, the best-scored poses were collected for the following MD simulation.

## Results and Discussion

### CoMFA and CoMSIA Model Analysis

**FIGURE 1:**
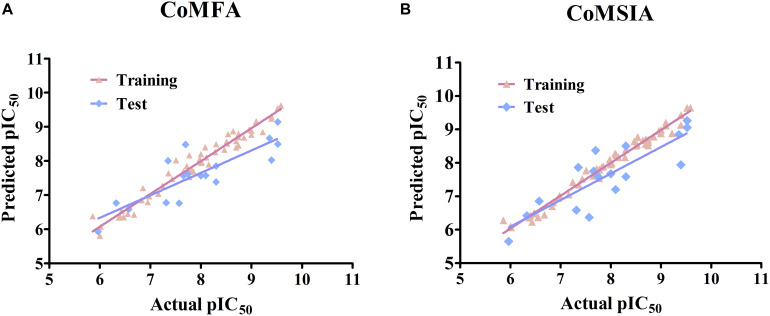
Linear fitting scatter plot of predicted activity values and experimental values for **(A)** the best CoMFA and **(B)** the best CoMSIA models.

Several CoMFA models were generated upon a series of 56 pyrrolopyrazine derivatives and the results are summarized in Supplementary Table S2. Besides the Tripos Standard force fields, another two “Advance CoMFA” fields including Indicator and H-bond were also applied for model generation. As we can see from Supplementary Table S2, the *q*^2^-values are all greater than 0.5, except for CoMFA(2). The *Region Focusing* module was then employed to refine the models by enhancing or attenuating the contribution of lattice points with the application of weights to these points (Zhu et al., 2012). After the implementation of region focusing, some models showed obvious improvements, especially CoMFA(6), and the results are shown in Table 1. CoMFA(6) with the steric and electrostatic fields contains the highest *q*^2^-value (0.711), and the other required parameters are all satisfactory: NOC = 8, SEE = 0.210, *r*^2^ = 0.963, *F* = 153.743. The contributions of steric and electrostatic fields are 61 and 39%, respectively, indicating that the activity of these inhibitors is governed more by the steric field. Then, a test set was used as an external dataset to evaluate the predictive ability of CoMFA(6), and CoMFA(6) showed good external predictability (*r*^2^*_*pred*_* = 0.713). In addition, the scatter plots were developed for the training and test data set using the experimental vs. predicted activity values, individually. The predicted pIC_50_ values for the training and test set, using CoMFA(6), are listed in Supplementary Table S1, and the linear correlation between predicted and experimental pIC_50_ of the training and test set is plotted in Figure 1A (*r*^2^*_*training*_* = 0.963 and *r*^2^*_*test*_* = 0.713). As shown in Figure 1A, most points are evenly distributed along the line Y = X, which demonstrates the good correspondence of the predicted model. The above results indicate that CoMFA(6) contains a reliable predictive capability for subsequent QSAR analysis.

**TABLE 1:** Detailed results of CoMFA models after the application of *Region Focusing*.

**Statistical parameters**		**CoMFA(6)**	**CoMFA(7)**	**CoMFA(8)**	**CoMFA(9)**	**CoMFA(10)**
*q*^2^		0.711	0.563	0.686	0.642	0.644
NOC		8	7	5	5	5
*r*^2^		0.963	0.901	0.883	0.891	0.889
SEE		0.210	0.341	0.363	0.351	0.353
F		153.743	62.154	75.174	81.338	79.968
**Field contributions**						
Tripos standard	S	0.610			0.330	
	E	0.390			0.207	
Indicator	S			0.801		0.368
	E			0.199		0.091
H-bond	A		0.868		0.361	0.428
	D		0.132		0.102	0.112

**q2, the cross-validated correlation; NOC, the optimum number of components; r^2^, the Non-cross-validated correlation; SEE, the standard error of estimation; F, F-test value; S, steric; E, electronic; A, acceptor; D, donor.**

Similar to CoMFA, in order to fully consider the effect of different fields, six CoMSIA models were constructed with different combinations of steric, electrostatic, hydrophobic, H-bond acceptor, and donor fields (Table 2). Among them, CoMSIA(4) with steric, electrostatic and hydrophobic fields caught our attention. As shown in Table 2, CoMSIA(4) possesses both the highest cross-validated correlation of 0.663, and also satisfactory values of *r*^2^, SEE, and F (0.982, 0.152, 238.862, respectively). The relevant contributions of steric, electrostatic, and hydrophobic fields are 18.7, 39.0, and 42.3%, respectively, which suggests that the electrostatic and hydrophobic features have a greater impact on the activity of the inhibitors. The external predictive ability of CoMSIA(4) was then also assessed, and the *r*^2^*_*pred*_* value reached 0.718. Similarly, the predicted activities of all inhibitors were calculated with CoMSIA(4) and the values are summarized in Supplementary Table S1, and the linear fitting plot of predicted vs. experimental pIC_50_ for training and test set is shown in Figure 1B (*r*^2^*_*training*_* = 0.982 and *r*^2^*_*test*_* = 0.718). As shown in Figure 1, CoMFA(6) and CoMSIA(4) both exhibit a robust predictive capability for subsequent analysis.

**TABLE 2:** Detailed results of CoMSIA models generated on several field combinations.

	***q*^2^**	**NOC**	***r*^2^**	**SEE**	**F**	**Field contribution**				
						**S**	**E**	**A**	**D**	**H**
CoMSIA(1)	0.606	9	0.971	0.188	171.762	0.367	0.633			
CoMSIA(2)	0.526	10	0.978	0.165	201.412	0.270	0.436	0.294		
CoMSIA(3)	0.596	10	0.976	0.172	186.235	0.322	0.537		0.141	
CoMSIA(4)	0.663	10	0.982	0.152	238.862	0.187	0.390			0.423
CoMSIA(5)	0.623	10	0.983	0.146	260.018	0.164	0.293	0.185		0.359
CoMSIA(6)	0.637	10	0.986	0.132	316.014	0.164	0.346		0.107	0.383
CoMSIA(7)	0.610	10	0.986	0.131	325.086	0.147	0.265	0.163	0.091	0.334

**q2, the cross-validated correlation; NOC, the optimum number of components; r^2^, the Non-cross-validated correlation; SEE, the standard error of estimation; F, F-test value; S, steric; E, electronic; A, acceptor; D, donor.**

### CoMFA and CoMSIA Contour Map Analysis

**FIGURE 2:**
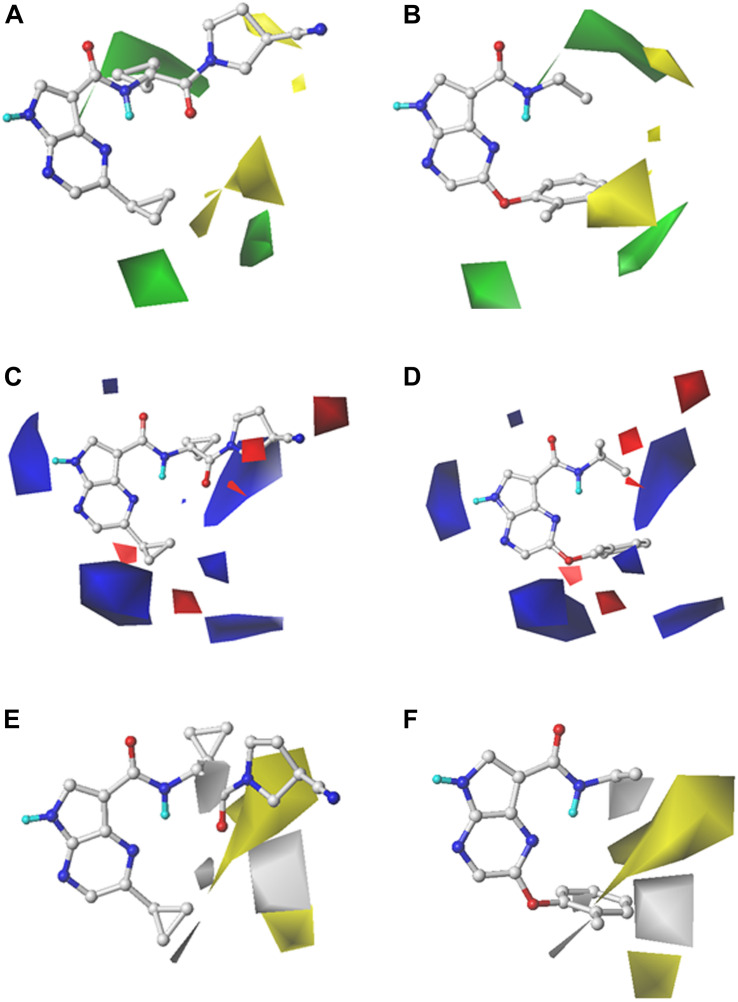
StDev*Coeff contour maps of the best CoMFA and CoMSIA models with Cpd16 and Cpd24. **(A,B)** Steric contour map of CoMFA with Cpd16 and Cpd24; **(C,D)** Electrostatic contour map of CoMFA with Cpd16 and Cpd24; **(E,F)** Hydrophobic contour map of CoMSIA with Cpd16 and Cpd24.

In order to reveal the SAR visually, the contour maps of CoMFA and CoMSIA models are illustrated in Figure 2. In order to facilitate the analysis and comparison, Cpd16 (pIC_50_ = 9.523) with the highest bioactivity and Cpd24 (pIC_50_ = 5.860) with the worst activity were embedded in the same contour map, respectively. The steric contour map of CoMFA is illustrated in Figures 2A,B, the green contour represents bulky groups and are associated with the enhancement of the activity, while the yellow one represents the contrary. First, the cyclopropyl attached to the C-atom between the bisamide of Cpd16 is covered by a green contour in Figure 2A, indicating that the introduction of bulky groups would be helpful to JAK3 potency. As expected, Cpd24 significantly decreases the JAK3 affinity when it lost this alkyl substituent (Figure 2B). Another two visible green contours below the pyrazine ring suggest that inhibitors with bulky substituents at this position would show better bioactivity. Take Cpd57 as an example, this compound contains a bigger group of benzpyrrole than the pyrazole of Cpd56 and shows a higher affinity to JAK3 (Supplementary Table S1). Additionally, the steric group should be located in the plane of the pyrropyrazine ring since a yellow block appears above the lower right green contour. In Figure 2B, the rotatable ether bond of Cpd24 led to the benzene ring moving out of the plane and staying closer to the yellow region, which sharply decreased the JAK3 inhibitory activity of Cpd24. Moreover, the same phenomenon could be observed in series Cpd18–27, which are all provided with an ether bond at the same place.

For the electrostatic fields, the blue (favorable) and red (unfavorable) contours represent the effect on the bioactivity when adding a positively or negatively charged group. At first glance in Figures 2C,D, the blue contours hold an absolute advantage, indicating that electropositive groups make favorable contributions to JAK3 potency. As shown in Figure 2C, there is a blue outline surrounding the electron-donating group -NH in the Cpd16, indicating that the presence of electron donor groups at that position is a guarantee of inhibitor activity. Another two giant blue blocks located near the two cyclopropyl motifs of Cpd16 (Figure 2C), suggest that positively charged groups in these regions are conducive for the promotion of the activity. For example, when the pendant cyclopropyl substituent attached to the pyrazine of Cpd1 (pIC_50_ = 7.352) was replaced by an electronegative group, such as the phenoxy group of Cpd18–27, a dramatic decline in activity by about 3–30 times appears (pIC_50_ range: 5.860–6.854). Similar, the introduction of electropositive groups to the position could improve the activity, such as Cpd58 (pIC_50_ = 8.959), Cpd67 (pIC_50_ = 9.398), and Cpd68 (pIC_50_ = 9.523) (Supplementary Table S1). In addition, two small blue contours at the lower-right side of the map manifest the increase of activity that would be obtained if an electropositive group was introduced. Thus Cpd58–69, bearing the electropositive benzopyrazole structure, shows relatively higher bioactivities. A red contour is observed near the -CN attached to the nitrogen heterocycle of Cpd16. It shows that placing a negative-charged group would be beneficial for JAK3 affinity (Figure 2C), while Cpd24 fails to fit the map because of the loss of this structural feature (Figure 2D).

As described above, the hydrophobic field of CoMSIA makes the greatest contribution to the inhibitor activity (42.3%). The hydrophobic contour map of CoMSIA(4) is illustrated in Figures 2E,F, and similarly, the Cpd16 and Cpd24 were both superposed over the contour maps. The yellow regions favor hydrophobic substituents and the white regions represent the opposite. As shown in Figure 2E, the pyrrolidine with a nitrile sidechain of Cpd16 is surrounded by a large yellow portion, suggesting that the hydrophobic interaction between inhibitors and JAK3 in the upper portion of the binding pocket would be favorable for JAK3 inhibition. However, Cpd24 fails to reach the yellow portion due to the loss of the nitrile pyrrolidine (Figure 2F). A similar effect will be obtained when truncating the pyrrolidine with a nitrile sidechain of Cpd13 (pIC50 = 8.086) to the dimethylamino of Cpd9 (pIC_50_ = 6.981) (Supplementary Table S1). Both Cpd58 (pIC_50_ = 8.959) and Cpd61 (pIC_50_ = 9.222) show higher activities than Cpd59 (pIC_50_ = 8.523) and Cpd60 (pIC_50_ = 8.538), which shows that a halogen substituent on the 6-position of the benzene ring may provide a favorable hydrophobic impact on JAK3 binding affinity (Supplementary Table S1). Furthermore, we can learn from the white block below in Figures 2E,F that a hydrophilic group here is desirable for JAK3 binding affinity. Nevertheless, as shown in Figure 2F, there is a hydrophobic phenyl group near the unfavorable hydrophobic white contour, which leads to the low activity of Cpd24. Cpd36 (pIC_50_ = 7.658) has similar pharmacophores with a hydrophilic sulfonamide group, it obtains higher activity than Cpd33 (pIC_50_ = 6.572), which loses a hydrophilic substituent on the ring. On the other hand, a small white block near the oxygen atom of Cpd16 is noticed in Figure 2E, and because of the hydrophilic amide oxygen and an extended bisamide, Cpd16 shows increasing activity than Cpd24.

### MD Simulation and Binding Free Energy Analysis

In order to estimate the dynamic behavior of JAK3 after binding to the selective inhibitors, a 200-ns MD simulation was carried out with two crystal complexes (Cpd10/JAK3, Cpd61/JAK3), respectively. The value of RMSD was calculated to monitor the relative deviation of backbone atoms and the results are illustrated in Supplementary Figure S2A. Overall, the RMSD values of both systems converged to 2.0 Å for the last 100 ns, which clearly showed that both systems reached equilibrium. To further explore the most favorable binding mechanism of the studied systems, the MM/GBSA method was used to evaluate the binding free energies for Cpd10 and Cpd61, and the results are tabulated in Table 3. The predicted binding free energies are −34.71 and −37.28 kcal/mol for Cpd10 (pIC_50_ = 7.162) and Cpd61 (pIC_50_ = 9.222), which is in good agreement with the experimental bioactivity. Comparing each energy contribution, van der Waals energy (ΔG_*VDW*_) plays a major role in JAK3 binding affinity, which are −45.34 kcal/mol for Cpd10 and −50.63 kcal/mol for Cpd61. That also confirms our QSAR analysis. For example, the nitrogen heterocycle with a nitrile sidechain and the benzene ring of Cpd61 fit well in the CoMSIA hydrophobic counter map. Furthermore, the contributions of the electrostatic interaction (ΔG_*ELE*_) is also noteworthy, which are −19.63 kcal/mol for Cpd10 and −25.33 kcal/mol for Cpd61. As with the QSAR results discussed above, the benzopyrazole and the nitrile pyrrolidine of Cpd61 fit better with the blue and red contours than the corresponding cyclopropyl and piperidine of Cpd10 in the CoMFA model (Supplementary Table S1).

**TABLE 3:** Binding free energies (kcal/mol) for JAK3-inhibitor complexes using the MM/GBSA method along with specific energy contributions.

**Compound**	**Δ G_*ELE*_^a^**	**Δ G_*VDW*_^b^**	**Δ G_*GB*_^c^**	**Δ G_*SA*_^d^**	**Δ G_*bind*_^e^**	**pIC_50_**
Cpd 10	−19.63 ± 0.95	−45.34 ± 0.71	34.04 ± 0.23	−3.78 ± 0.24	−34.71 ± 0.04	7.162
Cpd 61	−25.33 ± 1.30	−50.63 ± 1.77	42.82 ± 2.46	−4.14 ± 0.33	−37.28 ± 0.21	9.222

**^*a*^Electrostatic energy. ^*b*^van der Waals energy. ^*c*^Polar solvation energies. ^*d*^Non-polar solvation energies.**

**FIGURE 3:**
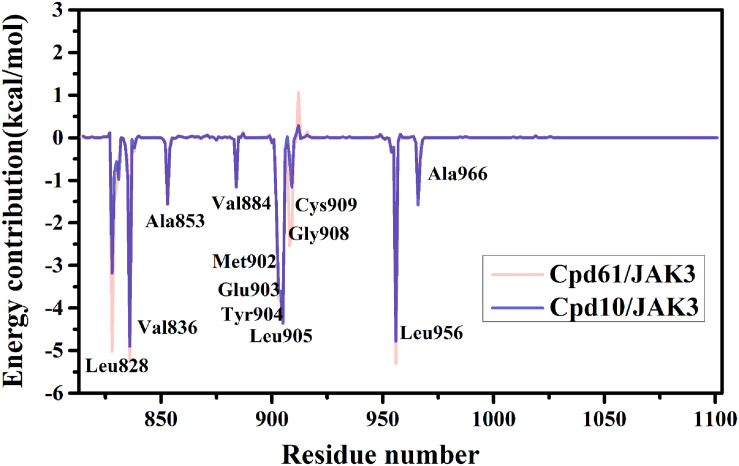
Plot of binding-free energy decomposition on per-residue for JAK3-inhibitor complexes.

**FIGURE 4:**
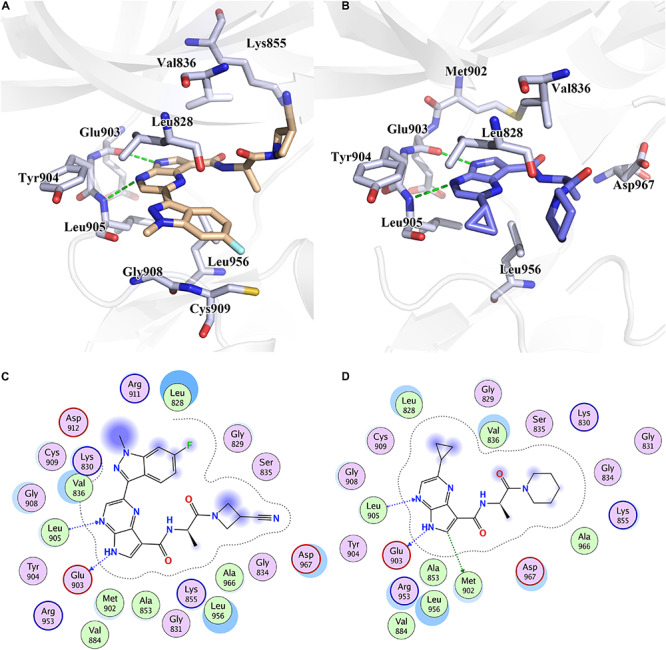
The binding pattern of **(A)** Cpd61 and **(B)** Cpd10 with JAK3. 2D mode interactions of **(C)** Cpd61 and **(D)** Cpd10 with JAK3.

Furthermore, to reveal the in-depth mechanism of the binding selectivity between JAK3 and the inhibitors, the free energy decomposition analysis between each inhibitor and JAK3 was calculated. The residue-inhibitor spectrums are plotted in Figure 3 and specific subdivisions of energy contributions of critical amino acid residues for the binding in the two systems are sorted in Supplementary Tables S3, S4. It is obvious that in both complexes, the residues surrounding the ATP binding pocket, such as Leu828, Val836, Glu903, Tyr904, Leu905, and Leu956, tend to yield more favorable interactions with the inhibitors (Figure 3). These residues within the catalytic pocket of JAK3 make the major contributions to total binding-free energy for both compounds. We first catch sight of the three hydrophobic residues, Leu828, Val836, and Leu956, because of their significant van der Waals interaction energy contributions. For Cpd10/JAK3, the ΔG_*VDW*_ of these residues are −3.68, −4.38, and −4.48 kcal/mol, and for Cpd61/JAK3, the values are −6.10, −4.74, and −4.94 kcal/mol (Supplementary Tables S3, S4). From Figure 4, we find that the common pyrrolopyrazine ring lies in this hydrophobic cavity composed of Leu828, Val836, and Leu956, leading to a stronger binding affinity to JAK3. Interestingly, This phenomenon can also be observed in the binding mode between Tofacitinib and JAK3 (Chrencik et al., 2010). As described above, the electrostatic interactions also show the favorable contribution to the binding free energies. As shown in Figure 4, there are two hydrogen bonds (H-bond) formed between pyrrolopyrazine ring and Glu903, Leu905 in the hinge region, with the distance of 2.8 and 3.1 Å, respectively. It is shown that Glu903 formed stronger ΔG_*ELE*_ with Cpd61 than Cpd10 (−12.06 kcal/mol vs. −10.82 kcal/mol), which may lead to a higher JAK3 inhibition for Cpd61. Therefore, the residues that formed strong H-bonds would play significant roles in JAK3 potency.

Although the two systems share resembling bindings and interactions, guaranteeing that the inhibitors could tightly bind with the receptor protein in its ATP-binding pocket, the existence of subtle discrepancies leads to the distinction on bioactivity. As shown in Figures 4A,C, compared with Cpd10, Cpd61 could pack the pocket better and facilitate the formation of shape in a complementary way. Firstly, these two compounds both contain a bisamide group, but the terminal –CN helps Cpd61 make a more in-depth filling and subsequently improves the activity, this is also in agreement with the CoMFA red contour around the –CN in Figure 2C. While for Cpd10, the loss of –CN and the steric effect of piperidine both prevent the pocket to fill deeply, it also leads to a further distance and a slightly weaker hydrophobic interaction with Val836 (Figure 4B). Secondly, the indazole group of Cpd61 orients toward the Cys909 in the front pocket, greatly facilitating the formation of favorable lipophilic interactions with Cys909, Gly908, and Leu828 (Figure 4A). It is also in accordance with our QSAR results which show that the huge hydrophobe indazole (Cpd61) could fit well with the yellow contour in the bottom right-hand corner of the CoMSIA hydrophobic contour map

(Figure 2E), while cyclopropyl (Cpd10) could not. In conclusion, these hot residues (Leu828, Val836, Glu903, Leu905, Cys909, and Leu956) are crucial for JAK3 inhibition and should be taken into account when designing new inhibitors against JAK3.

### New Inhibitors Design

Combining the suggestions derived from 3D-QSAR maps and the binding mechanisms explored by the MD simulation, 10 analogous JAK3 inhibitors were designed based on Cpd61. According to the SAR described above, the pyrrolopyrazine core-scaffold was retained to maintain the key H-bond interactions, and the nitrile side chain was also retained because molecules with such structural features could fill the back and upper binding pocket to improve binding affinity. Subsequently, the *De Novo* protocol in DS 3.5 was employed to produce new analogous inhibitors based on the nitrile-pyrrolopyrazine scaffold. The structures of 10 newly-designed inhibitors are listed in Table 4. They were all sketched and optimized in SYBYL-X 2.0 in the same way as mentioned above and their activities were predicted by the CoMFA(6) and CoMSIA(4) models (Table 4). Among them, D9 exhibited the highest predicted binding affinity with JAK3.

**TABLE 4:** The 2D structures, predicted activities of 10 novel JAK3 inhibitors.

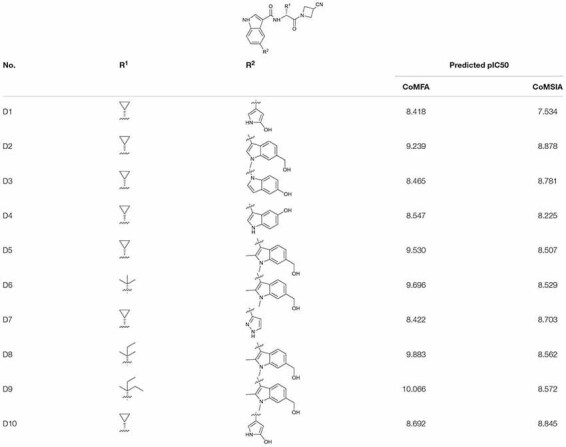				

### JAK3 Selective Mechanisms of the New Designed Compound

In order to verify the accuracy and reliability of the QSAR models and to investigate whether the obtained SAR results can be used to guide the design of novel JAK3 selective inhibitors, D9, with highest predictive activity was chosen to show the JAK3 potency and selectivity through the MD simulation and binding free energy calculations. Specifically, a 200-ns MD was subjected on three JAK isoform complexes, namely, D9/JAK1 (PDB: 6N7A), D9/JAK2 (PDB: 4IVA), and D9/JAK3 (PDB: 4HVI). As shown in Supplementary Figure S2B, three systems reached equilibrium after 200 ns. The binding free energies and decomposition of the three systems were then calculated using the MM/GBSA method and the results are listed in Table 5. The binding affinity of D9/JAK3 is the highest and is in agreement with the predictive activity of QSAR models (D9/JAK1: −33.92 kcal/mol; D9/JAK2: −29.12 kcal/mol; D9/JAK3: −48.92 kcal/mol), indicating that D9 prefers to selectively inhibit JAK3 compared to the other two JAK isoforms. According to Table 5, ΔG_*ELE*_ significantly contributes to the selective binding for D9 to JAK3 (D9/JAK1: −81.14 kcal/mol; D9/JAK2: −63.39 kcal/mol; D9/JAK3: −116.37 kcal/mol). In addition, the ΔG_*ELE*_ value of D9/JAK3 is almost two fold over those of D9/JAK2 and D9/JAK1, so the electrostatic interaction is predominantly responsible for the highest JAK3 inhibitory potency of D9. Although the values of ΔG_*VDW*_ are overall lower than ΔG_*ELE*_ in the three systems and their distinction of ΔG_*VDW*_ is not very large (D9/JAK1: −53.64 kcal/mol; D9/JAK2: −51.41 kcal/mol; D9/JAK3: −60.91 kcal/mol), it may still be a driver of D9 binding to JAK3 preferentially.

**TABLE 5:** Binding free energies (kcal/mol) for D9 with JAKs using the MM/GBSA method along with specific energy contributions.

**Complex**	**Δ G_*ELE*_^a^**	**Δ G_*VDW*_^b^**	**Δ G_*GB*_^c^**	**Δ G_*SA*_^d^**	**Δ G_*bind*_^e^**
D9/JAK1	−81.14 ± 3.89	−53.64 ± 1.84	105.33 ± 2.36	−4.48 ± 0.39	−33.92 ± 1.23
D9/JAK2	−63.39 ± 2.92	−51.41 ± 0.54	89.81 ± 2.64	−4.13 ± 0.29	−29.12 ± 1.33
D9/JAK3	−116.37 ± 1.56	−60.91 ± 0.04	133.83 ± 1.62	−5.46 ± 0.26	−48.92 ± 1.51

**^*a*^Electrostatic energy. ^*b*^van der Waals energy. ^*c*^Polar solvation energies. ^*d*^Non-polar solvation energies.**

**FIGURE 5:**
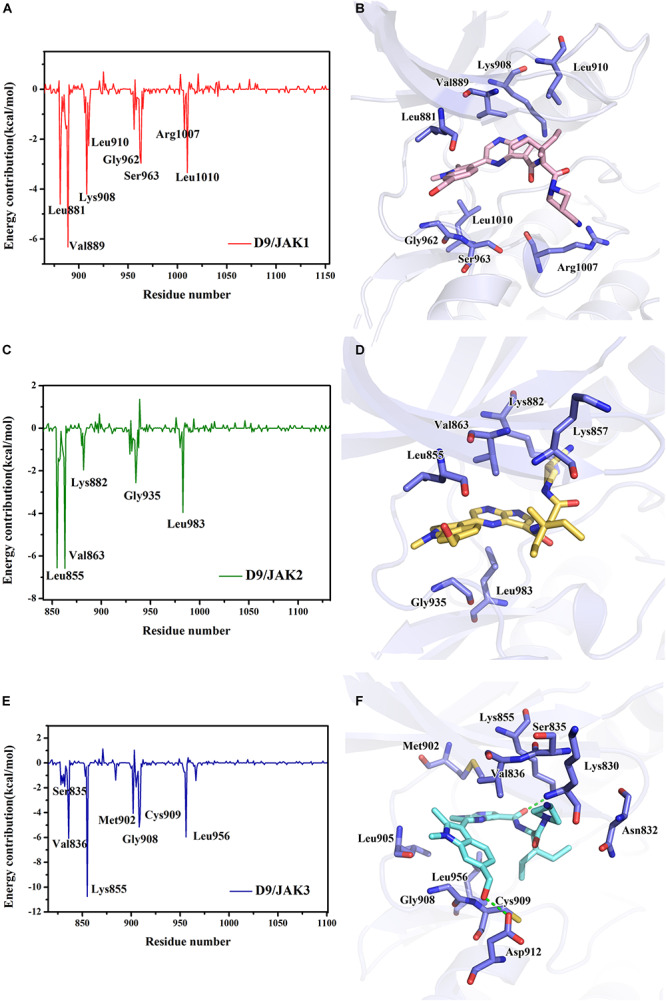
The plots of binding-free energy decomposition on per-residue for **(A)** D9/JAK1, **(C)** D9/JAK2, **(E)** D9/JAK3. The binding patterns of D9 in the active domain of **(B)** JAK1, **(D)** JAK2 and **(F)** JAK3.

**FIGURE 6:**
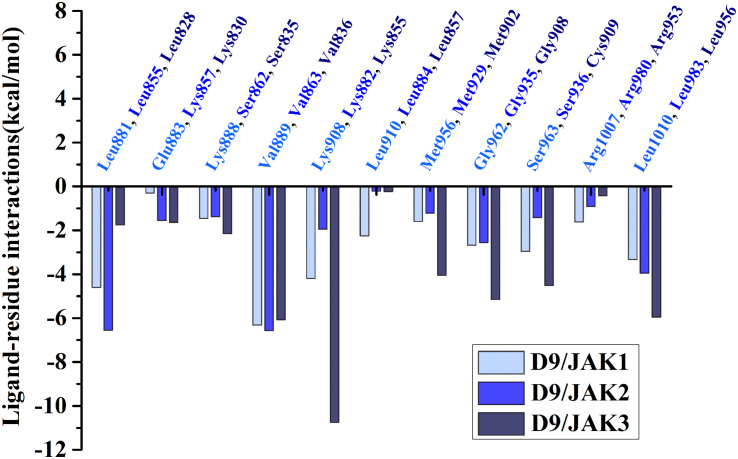
Comparison of energy contributions of the important corresponding residues for the D9/JAK1 (wathet), D9/JAK2 (blue), D9/JAK3 (navy).

Subsequently, the binding energies of the three systems were broken up into the energy contribution on each residue. As shown in Figure 5, critical residues that were highly involved in the binding process of JAK-inhibitors have been annotated and the MD-simulated binding patterns are all displayed (Supplementary Tables S5–S7). Additionally, as a function during the MD simulation, H-bond occupancy analyzation was performed. For each system, the last 100 ns of the MD trajectory was split into 1,000 intervals and the H-bond occupancy was calculated for each interval. The results are tallied and displayed in Table 6. First, during the dynamic simulation of D9/JAK3, an extraordinarily strong H-bond between the −OH in benzyl alcohol of D9 and Asp912 was formed (occupancy up to 63.41%, Figure 5F and Table 6), while this H-bond was both lost in the other JAK isoforms (Glu966/JAK1 = 2.33% and Asp939/JAK2 = 0.03%, Figures 5B,D). As shown in Figure 5F, the R^2^ group of D9 rotates, making the OH closer to Asp912 of JAK3 to form a strong H-bond interaction. This strong H-bond also induces the stronger VDW contacts between the R^2^ group and Leu881, Val889 of JAK1, or Leu855, Val863 of JAK2 (Figures 5B,D). Thus, the introduction of a strong H-bond between Asp912 and inhibitors may impart a large degree of selectivity for JAK3. Another strong H-bond was formed between D9 and Lys855 of JAK3 and the occupancy is significantly higher than other corresponding residues in other JAK isoforms (53.64% for Lys855/JAK3, 2.6% for Lys908/JAK1 and 2.04% for Lys882/JAK2, Table 6), The superimposed conformation comparison of the three complexes in Supplementary Figure S3 showed that the carbonyl oxygen of the ligand orients upward to the Lys855, while in D9/JAK1 or D9/JAK2, this carbonyl oxygen is in the reverse direction so that they fail to form the H-bond at this position. Based on the conformation comparison, we hypothesize that the hydrophobic effect of the lower Leu956 is the key driver pushing the carbonyl oxygen up, thereby forming this H-bond interaction. As shown in Figure 6, the ΔG_*VDW*_ of Leu956 is higher than that of corresponding Leu1010/JAK1 and Leu983/JAK2. In addition, an H-bond was also formed between D9 and Cys909 of JAK3 (11.14%, Figure 5F), and it is also higher than Ser963/JAK1 (2.4%) and Ser936/JAK2 (0%). Furthermore, the specific Cys909 of JAK3 also formed a more favorable hydrophobic contact with the R^2^ substituent of D9 than that in Ser963/JAK1 and Ser936/JAK2 (Supplementary Figure S4), consistent with the CoMSIA results discussed above. Thus, it is a crux accounting for the highest JAK3 binding affinity (Lynch et al., 2013).

**TABLE 6:** The analysis of the H-bonds occupancy during the MD simulation process for D9/JAKs.

**Complex**	**Donor**	**Acceptor**	**Occupancy (%)**
D9/JAK1	D9	Leu881	24.23 ± 13.86
	Lys908	D9	2.60 ± 3.11
	Ser963	D9	2.40 ± 3.25
	D9	Glu966	2.33 ± 10.64
D9/JAK2	D9	Leu855	34.10 ± 8.38
	Lys882	D9	2.04 ± 2.99
	D9	Asp939	0.03 ± 0.00
D9/JAK3	D9	Asp912	63.41 ± 25.25
	Lys855	D9	53.64 ± 7.27
	Cys909	D9	11.14 ± 5.60
	Asn832	D9	7.33 ± 7.23

*The occupancy of the H-bonds was calculated based on the last 100 ns of the MD trajectory using VMD software. The uncertainties of the occupancies were defined as $\text{SD}=\sqrt{\frac{1}{n}\,\sum_{i=0}^{n}{\left(x_{i}-\bar{X}\right)}^{2}}$.

## Conclusion

In this study, a series of JAK3 inhibitors were collected and several molecular modeling studies, including 3D-QSAR, MD simulation, free energy calculation, and decomposition, were carried out to investigate the selective binding mechanism of JAK3. First, two reliable CoMFA and CoMSIA models were built and the results explained the relationship between the structures and the JAK3 binding activities well. Then, two representative JAK3-inhibitor crystal structures were subjected to MD simulation, and several key residues relating to high activity and selectivity were highlighted after free energy calculation and decomposition. Based on the results of QSAR and MD simulations, 10 new compounds with the same skeleton were designed, the bioactivities were predicted through CoMFA and CoMSIA models, and they all showed high predicted activities, especially D9. Finally, three JAK isoforms/D9 complexes were subjected to MD simulation, and D9 showed the highest selective inhibition to JAK3, suggesting that our studies successfully reveal the selective mechanisms of JAK3 inhibition and may provide significant guidance in the design of novel selective JAK3 inhibitors.

## Data Availability Statement

All datasets generated for this study are included in the article/Supplementary Material.

## Author Contributions

JZ and JJ developed the study concept and design. QY performed the studies and carried out the data analysis. JZ, QY, and YC drafted the manuscript. HL provided software. WL, YC, and JJ approved the manuscript.

## Conflict of Interest

WL was employed by the company CF PharmTech. The remaining authors declare that the research was conducted in the absence of any commercial or financial relationships that could be construed as a potential conflict of interest.
