# Supplementary material for: Theoretical Exploring Selective-Binding Mechanisms of JAK3 by 3D-QSAR, Molecular Dynamics Simulation and Free Energy Calculation
Source: Front Mol Biosci. 2020 May 27;7:83. doi: 10.3389/fmolb.2020.00083 (PMC7266956; doi:10.3389/fmolb.2020.00083)
Supplement: Supplementary file 1 [file Data_Sheet_1.docx]

# Supplemental Materials


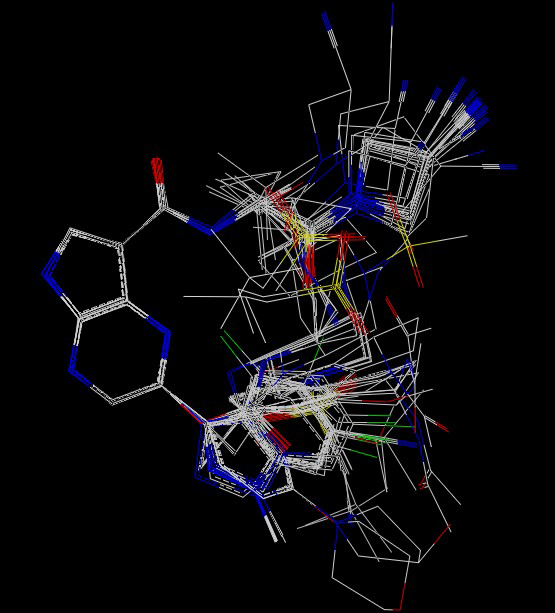


**Figure S1.** The alignment of the inhibitors based on the common core-pyrrolopyrazine.

**
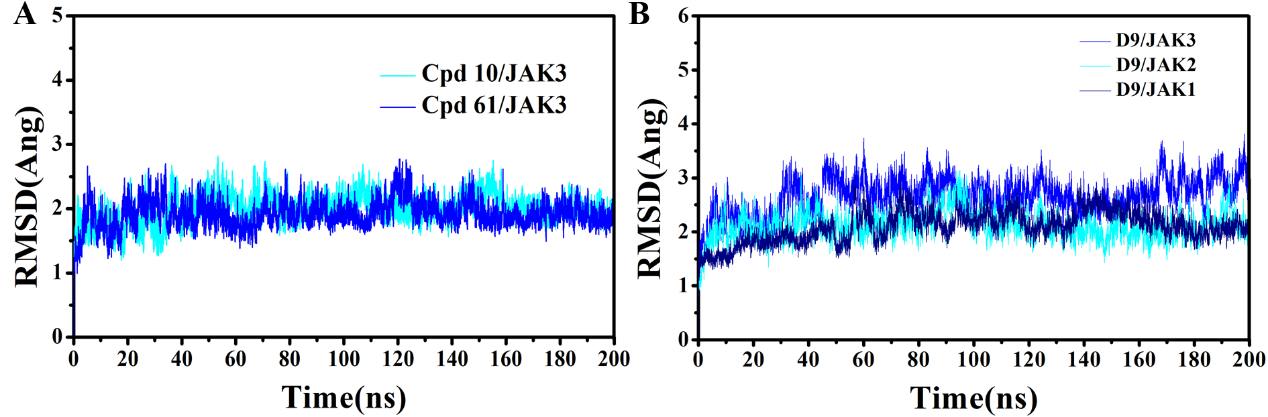
**

**Figure S2.** The root-mean-square deviation (RMSD) of atomic locations of **(A)** Cpd61/10-JAK3 complexes and **(B)** D9-JAKs complexes.

**
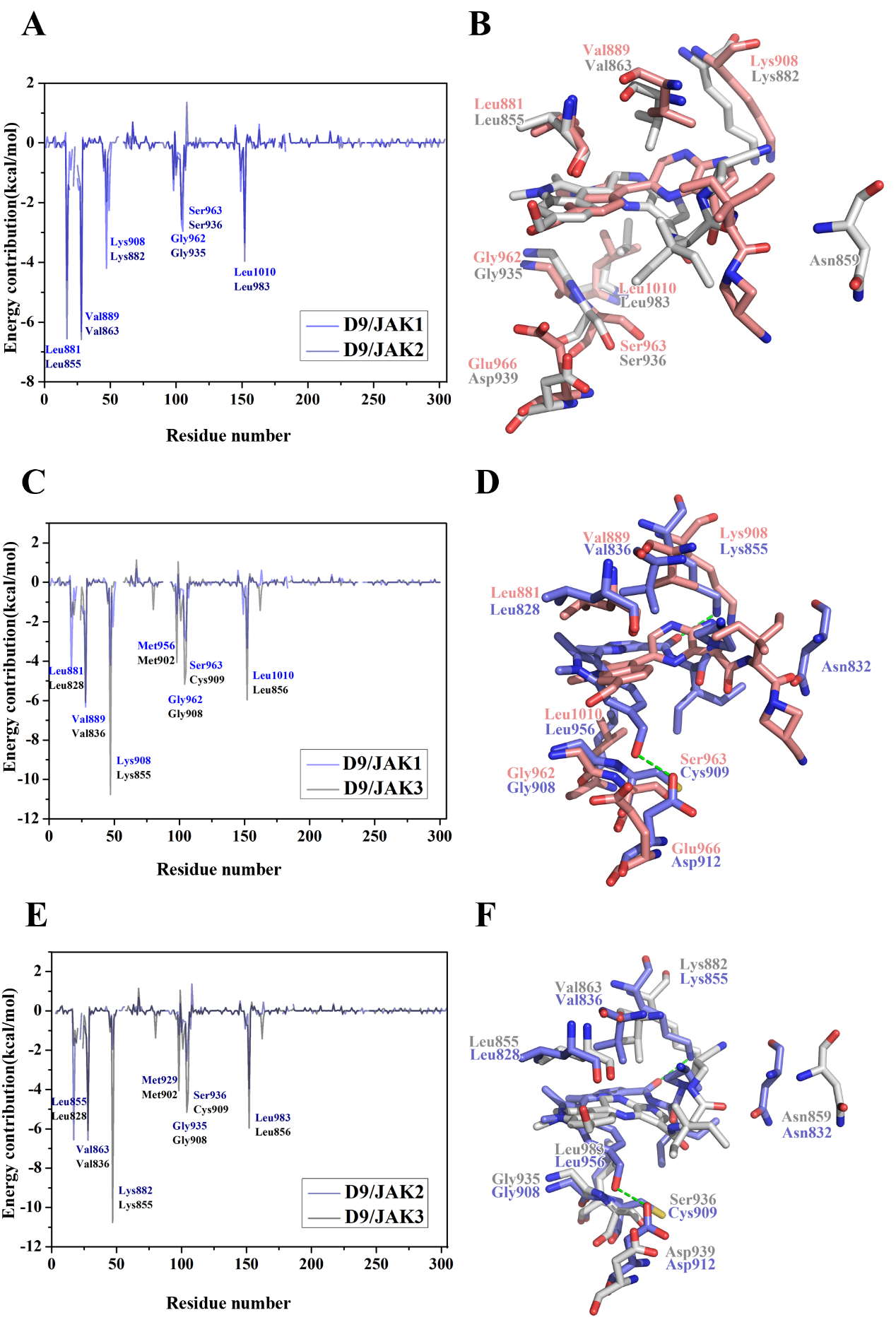
**

**Figure S3. (A, C, E)** The D9-residues overlapped interaction spectrums (blue: D9-JAK1, navy: D9-JAK2, black: D9-JAK3); **(B, D, F)** D9-JAKs overlapped binding pattern (red: D9-JAK1, silvery: D9-JAK2, blue: D9-JAK3)


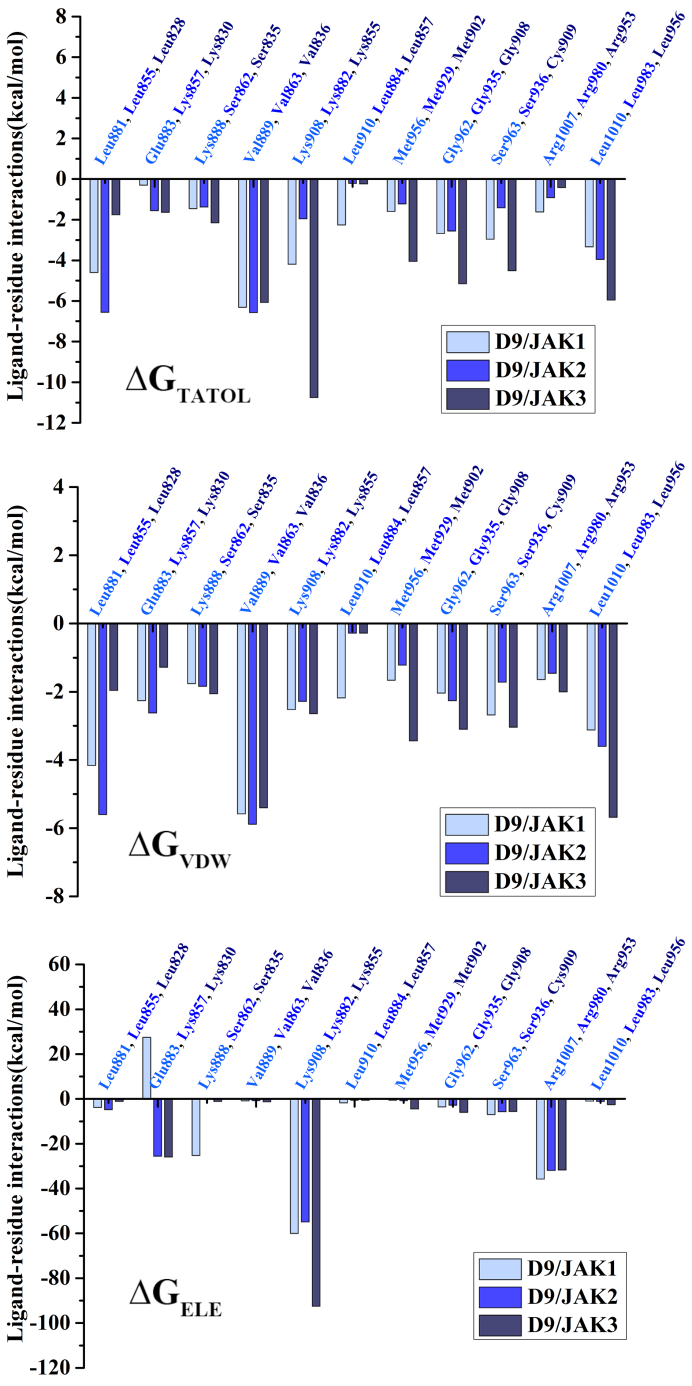


**Figure S4.** Comparison of energy contributions of the important corresponding residues for the D9/JAK1 (wathet), D9/JAK2 (blue), D9/JAK3 (navy).

**Table S1** Structures of JAK3 inhibitors with their experimental and predicted (pIC_50_)

(* represents compounds in test set)

|  | | | | | | | | |
| --- | --- | --- | --- | --- | --- | --- | --- | --- |
| Compound | R | | Actual IC_50_(nM) | | Actual pIC_50_ | | Predicted pIC_50_ | |
|  |  |  |  |  |  |  | CoMFA | CoMSIA |
| 1^*^ |  | | 44.5 | | 7.352 | | 8.004 | 7.862 |
| 2 |  | | 9.6 | | 8.018 | | 7.966 | 7.991 |
| 3 |  | | 7.8 | | 8.108 | | 8.107 | 8.231 |
| 4 |  | | 1.4 | | 8.854 | | 8.686 | 8.787 |
| 5 |  | | 17.0 | | 7.770 | | 8.169 | 7.804 |
| 6 |  | | 19.0 | | 7.721 | | 7.85 | 7.824 |
| 7 |  | | 1.8 | | 8.745 | | 8.458 | 8.52 |
| 8 |  | | 43.6 | | 7.361 | | 7.641 | 7.344 |
| 9 |  | | 104.4 | | 6.981 | | 6.962 | 7.014 |
| 10 |  | | 68.9 | | 7.162 | | 7.045 | 7.052 |
| 11^*^ |  | | 48.5 | | 7.314 | | 6.776 | 6.586 |
| 12 |  | | 148.0 | | 6.830 | | 6.85 | 6.701 |
| 13 |  | | 8.2 | | 8.086 | | 8.399 | 8.281 |
| 14 |  | | 0.26 | | 9.585 | | 9.631 | 9.597 |
| 15 |  | | 0.4 | | 9.398 | | 9.312 | 9.214 |
| 16 |  | | 0.3 | | 9.523 | | 9.556 | 9.651 |
| 17^*^ |  | | 0.44 | | 9.356 | | 8.667 | 8.849 |
|  | | | | | | | | |
| Compound | R^1^ | | R^2^ | | Actual IC_50_(nM) | Actual pIC_50_ | Predicted pIC_50_ | |
|  |  |  |  |  |  |  | CoMFA | CoMSIA |
| 18 | -Me | | -H | | 210 | 6.678 | 6.426 | 6.444 |
| 19 | -Me | | m-Me | | 280 | 6.553 | 6.453 | 6.371 |
| 20^*^ | -Me | | m-Et | | 470 | 6.328 | 6.768 | 6.418 |
| 21 | -Me | | 3,5-di-OMe | | 400 | 6.398 | 6.388 | 6.425 |
| 22 | -Me | | m-CN | | 970 | 6.013 | 6.074 | 6.071 |
| 23 | -Me | | p-CN | | 420 | 6.377 | 6.339 | 6.364 |
| 24 | -Me | | o-Me | | 1380 | 5.860 | 6.38 | 6.281 |
| 25 |  | | | | 370 | 6.432 | 6.544 | 6.23 |
| 26 |  | | | | 340 | 6.468 | 6.352 | 6.478 |
| 27 |  | | | | 140 | 6.854 | 7.204 | 6.816 |
| 28^*^ |  | | | | 22 | 7.658 | 7.564 | 7.749 |
| 29 |  | | | | 14 | 7.854 | 7.69 | 7.896 |
| 30 |  | | | | 112 | 6.951 | 6.791 | 6.951 |
| 31^*^ |  | | | | 5 | 8.301 | 7.386 | 7.584 |
| 32 |  | | | | 57 | 7.244 | 7.275 | 7.424 |
|  | | | | | | | | |
| Compound | R | | Actual IC_50_(nM) | | Actual pIC_50_ | | Predicted pIC_50_ | |
|  |  |  |  |  |  |  | CoMFA | CoMSIA |
| 33^*^ |  | | 268 | | 6.572 | | 6.585 | 6.857 |
| 34 |  | | 1000 | | 6.000 | | 5.813 | 6.098 |
| 35^*^ |  | | 1083 | | 5.965 | | 5.932 | 5.649 |
| 36 |  | | 22 | | 7.658 | | 7.7 | 7.611 |
| 37 |  | | 10 | | 8.000 | | 7.941 | 7.952 |
| 38 |  | | 7 | | 8.155 | | 7.896 | 8.163 |
| 39 |  | | 2 | | 8.699 | | 8.398 | 8.71 |
| 40^*^ |  | | 5 | | 8.301 | | 7.851 | 8.504 |
| 41 |  | | 334 | | 6.476 | | 6.353 | 6.5 |
| 42 |  | | | 31 | 7.509 | | 8.023 | 7.767 |
| 43^*^ |  | | | 27 | 7.569 | | 6.76 | 6.37 |
| 44 |  | | | 37 | 7.432 | | 7.474 | 7.519 |
| 45^*^ |  | | | 20 | 7.699 | | 8.484 | 8.372 |
| 46 |  | | | 2 | 8.699 | | 8.579 | 8.561 |
| 47 |  | | | 1 | 9.000 | | 8.889 | 8.884 |
| 48 |  | | | 5 | 8.301 | | 8.489 | 8.406 |
|  | | | | | | | | |
| Compound | R | | Actual IC_50_(nM) | | Actual pIC_50_ | | Predicted pIC_50_ | |
|  |  |  |  |  |  |  | CoMFA | CoMSIA |
| 49^*^ | -NH2 | | 18 | | 7.745 | | 7.614 | 7.589 |
| 50 | -OH | | 17 | | 7.770 | | 7.53 | 7.534 |
| 51^*^ |  | | 8 | | 8.097 | | 7.575 | 7.202 |
| 52 |  | | 15 | | 7.824 | | 7.729 | 7.88 |
| 53 |  | | 18 | | 7.745 | | 7.749 | 7.716 |
| 54^*^ |  | | 10 | | 8.000 | | 7.568 | 7.676 |
|  | | | | | | | | |
| Compound | R | | Actual IC_50_(nM) | | Actual pIC_50_ | | Predicted pIC_50_ | |
|  |  |  |  |  |  |  | CoMFA | CoMSIA |
| 55^*^ |  | | 0.4 | | 9.398 | | 8.027 | 7.94 |
| 56 |  | | 5.2 | | 8.284 | | 8.261 | 8.167 |
| 57 |  | | 0.8 | | 9.097 | | 9.163 | 9.113 |
|  | | | | | | | | |
| Compound | R^1^ | R^2^ | Actual IC_50_(nM) | | Actual pIC_50_ | | Predicted pIC_50_ | |
|  |  |  |  |  |  |  | CoMFA | CoMSIA |
| 58 |  | 6-Cl | 1.1 | | 8.959 | | 8.924 | 8.955 |
| 59 |  | 5-Cl | 3.0 | | 8.523 | | 8.513 | 8.549 |
| 60 |  | H | 2.9 | | 8.538 | | 8.787 | 8.771 |
| 61^*^ |  | 6-F | 0.6 | | 9.222 | | 8.846 | 8.885 |
| 62 |  | 6-CN | 2.3 | | 8.638 | | 8.875 | 8.67 |
| 63 |  | 6-OMe | 1.8 | | 8.745 | | 8.795 | 8.697 |
| 64 |  | 6-Cyclo-Pr | 3.1 | | 8.509 | | 8.564 | 8.515 |
| 65 |  | 6-t-Bu | 10 | | 8.000 | | 8.204 | 7.958 |
| 66 |  | 4-F,6-Cl | 1.8 | | 8.745 | | 8.699 | 8.623 |
| 67 |  | 6-Cl | 0.4 | | 9.398 | | 9.243 | 9.432 |
| 68^*^ |  | 6-Cl | 0.3 | | 9.523 | | 8.146 | 9.261 |
| 69 |  | 6-Cl | 0.8 | | 9.097 | | 9.146 | 9.206 |
|  | | | | | | | | |
| Compound | R | | Actual IC_50_(nM) | | Actual pIC_50_ | | Predicted pIC_50_ | |
|  |  |  |  |  |  |  | CoMFA | CoMSIA |
| 70 |  | | 10.5 | | 7.979 | | 8.063 | 8.099 |
| 71^*^ |  | | 0.3 | | 9.523 | | 8.496 | 9.057 |
| 72 |  | | 1.0 | | 9.000 | | 8.774 | 8.982 |
| 73 |  | | 3.4 | | 8.469 | | 8.328 | 8.641 |

**Table S2.** Detailed results of CoMFA models generated on several field combinations

| Statistical parameters | | CoMFA(1) | CoMFA(2) | CoMFA(3) | CoMFA(4) | CoMFA(5) |
| --- | --- | --- | --- | --- | --- | --- |
| *q^2^* | | 0.680 | 0.492 | 0.661 | 0.608 | 0.596 |
| NOC | | 6 | 5 | 5 | 7 | 6 |
| *r^2^* | | 0.930 | 0.880 | 0.892 | 0.922 | 0.924 |
| SEE | | 0.283 | 0.368 | 0.348 | 0.299 | 0.296 |
| F | | 108.727 | 73.096 | 82.744 | 96.636 | 98.729 |
| Field contribution | | | | | | |
| Tripos standard | S | 0.557 |  |  | 0.296 |  |
|  | E | 0.443 |  |  | 0.231 |  |
| Indicator | S |  |  | 0.804 |  | 0.372 |
|  | E |  |  | 0.196 |  | 0.115 |
| H-bond | A |  | 0.804 |  | 0.351 | 0.413 |
|  | D |  | 0.196 |  | 0.122 | 0.101 |

*q^2^*: the cross-validated correlation; NOC: the optimum number of components; *r^2^*; the Non-cross-validated correlation; SEE: the standard error of estimation; F: F-test value. S: steric; E: electronic; A: acceptor; D: donor.

**Table S3.** The energy contributions of key residues for the binding of Cpd61/JAK3 (kcal/mol)

| **Residue** | **ΔG_VDW_** | **ΔG_ELE_** | **ΔG_GB_** | **ΔG_SA_** | **ΔG_total_** |
| --- | --- | --- | --- | --- | --- |
| **Leu828** | -6.1 | -0.26 | 2.24 | -0.9 | -5.02 |
| Gly829 | -1.94 | -0.62 | 1.4 | -0.26 | -1.44 |
| Lys830 | -1.38 | -2.6 | 2.94 | -0.16 | -1.18 |
| **Val836** | -4.74 | 0.02 | 0.1 | -0.62 | -5.26 |
| **Ala853** | -1.62 | -0.1 | 0.24 | -0.08 | -1.58 |
| Lys855 | -1.24 | -4.62 | 5.9 | -0.12 | -0.08 |
| **Val884** | -1.18 | 0.52 | -0.42 | -0.06 | -1.14 |
| Met902 | -2.08 | 0.78 | -0.14 | -0.16 | -1.6 |
| **Glu903** | -0.02 | -12.06 | 8.34 | -0.04 | -3.76 |
| **Tyr904** | -3 | -2.76 | 2.02 | -0.1 | -3.86 |
| **Leu905** | -3 | -3.28 | 2.2 | -0.14 | -4.22 |
| Gly908 | -2.5 | -0.88 | 1.18 | -0.36 | -2.54 |
| Cys909 | -2.42 | -0.1 | 0.56 | -0.3 | -2.28 |
| Asn954 | -1.06 | -0.42 | 1.18 | -0.08 | -0.36 |
| **Leu956** | -4.94 | 0.06 | 0.18 | -0.6 | -5.3 |
| **Ala966** | -1.26 | -1.06 | 1.02 | -0.1 | -1.4 |
| Asp967 | -2.14 | -1.88 | 4.24 | -0.4 | -0.18 |

**Table S4.** The energy contributions of key residues for the binding of Cpd10/JAK3 (kcal/mol)

| **Residue** | **ΔG_VDW_** | **ΔG_ELE_** | **ΔG_GB_** | **ΔG_SA_** | **ΔG_total_** |
| --- | --- | --- | --- | --- | --- |
| **Leu828** | -3.68 | -0.06 | 1.14 | -0.58 | -3.18 |
| Gly829 | -1.6 | -0.2 | 1.16 | -0.18 | -0.82 |
| Lys830 | -1.58 | -0.12 | 1.3 | -0.16 | -0.56 |
| **Val836** | -4.38 | -0.36 | 0.4 | -0.56 | -4.9 |
| **Ala853** | -1.6 | -0.16 | 0.3 | -0.1 | -1.56 |
| Lys855 | -1.64 | -2.44 | 4.28 | -0.2 | 0 |
| **Val884** | -1.16 | 0.4 | -0.34 | -0.06 | -1.16 |
| **Met902** | -2.04 | 0.66 | 0 | -0.18 | -1.54 |
| **Glu903** | 0.1 | -10.82 | 7.12 | -0.04 | -3.64 |
| **Tyr904** | -2.72 | -2.48 | 1.66 | -0.06 | -3.6 |
| **Leu905** | -2.92 | -4.08 | 2.78 | -0.14 | -4.36 |
| Gly908 | -1.12 | 0.74 | 0.12 | -0.22 | -0.48 |
| Cys909 | -1.14 | -0.2 | 0.4 | -0.2 | -1.16 |
| **Leu956** | -4.48 | 0 | 0.28 | -0.6 | -4.78 |
| **Ala966** | -1.42 | -1.3 | 1.26 | -0.12 | -1.58 |
| Asp967 | -2.7 | -2.62 | 5.34 | -0.52 | -0.48 |

**Table S5.** The energy contributions of key residues for the binding of D9/JAK1 (kcal/mol)

| **Residue** | **ΔG_VDW_** | **ΔG_ELE_** | **ΔG_GB_** | **ΔG_SA_** | **ΔG_total_** |
| --- | --- | --- | --- | --- | --- |
| Leu881 | -4.16 | -3.8 | 4.02 | -0.68 | -4.6 |
| Val889 | -5.58 | -0.84 | 0.86 | -0.76 | -6.32 |
| Lys908 | -2.52 | -59.96 | 58.58 | -0.3 | -4.2 |
| Leu910 | -2.18 | -1.74 | 1.92 | -0.26 | -2.26 |
| Gly962 | -2.04 | -3.54 | 3.22 | -0.32 | -2.68 |
| Ser963 | -2.68 | -6.98 | 7.12 | -0.44 | -2.96 |
| Arg1007 | -1.64 | -35.72 | 36.12 | -0.38 | -1.62 |
| Leu1010 | -3.12 | -0.98 | 1.22 | -0.48 | -3.34 |
| Leu881 | -4.16 | -3.8 | 4.02 | -0.68 | -4.6 |

**Table S6.** The energy contributions of key residues for the binding of D9/JAK2 (kcal/mol)

| **Residue** | **ΔG_VDW_** | **ΔG_ELE_** | **ΔG_GB_** | **ΔG_SA_** | **ΔG_total_** |
| --- | --- | --- | --- | --- | --- |
| Leu855 | -5.6 | -4.76 | 4.78 | -0.96 | -6.56 |
| Lys857 | -2.62 | -25.52 | 26.84 | -0.26 | -1.56 |
| Val863 | -5.88 | -0.76 | 0.78 | -0.7 | -6.58 |
| Lys882 | -2.28 | -54.88 | 55.56 | -0.36 | -1.96 |
| Gly935 | -2.26 | -2.82 | 2.86 | -0.34 | -2.56 |
| Ser936 | -1.72 | -5.7 | 6.36 | -0.38 | -1.42 |
| Leu983 | -3.6 | -1.14 | 1.38 | -0.62 | -3.96 |

**Table S7.** The energy contributions of key residues for the binding of D9/JAK3 (kcal/mol)

| **Residue** | **ΔG_VDW_** | **ΔG_ELE_** | **ΔG_GB_** | **ΔG_SA_** | **ΔG_total_** |
| --- | --- | --- | --- | --- | --- |
| Lys830 | -1.28 | -25.94 | 25.68 | -0.08 | -1.64 |
| Asn832 | -2.94 | -0.62 | 2.22 | -0.54 | -1.9 |
| Ser835 | -2.06 | -1.14 | 1.08 | -0.04 | -2.16 |
| Val836 | -5.4 | -1.28 | 1.3 | -0.7 | -6.08 |
| Lys855 | -2.64 | -92.54 | 85.28 | -0.84 | -10.76 |
| Met902 | -3.44 | -4.48 | 4.22 | -0.36 | -4.06 |
| Leu905 | -3.84 | 0.44 | 1.74 | -0.3 | -1.94 |
| Gly908 | -3.1 | -5.98 | 4.26 | -0.36 | -5.16 |
| Cys909 | -3.04 | -5.58 | 4.48 | -0.38 | -4.52 |
| Leu956 | -5.68 | -2.52 | 2.92 | -0.68 | -5.96 |
